# Long-term changes in the planktonic cnidarian community in a mesoscale area of the NW Mediterranean

**DOI:** 10.1371/journal.pone.0196431

**Published:** 2018-05-01

**Authors:** Elena Guerrero, Josep-Maria Gili, Jordi Grinyó, Vanesa Raya, Ana Sabatés

**Affiliations:** Institut de Ciències del Mar, CSIC, Barcelona, Spain; University of Shiga Prefecture, JAPAN

## Abstract

In the present work, possible long-term changes in the planktonic cnidarian community were investigated by analyzing (1) species and community spatial distribution patterns, (2) variations in abundance and (3) changes in species richness during three mesoscale surveys representative of the climatic and anthropogenic changes that have occurred during the last three decades (years: 1983, 2004 and 2011) in the NW Mediterranean. These surveys were conducted during the summer (June) along the Catalan coast. All surveys covered the same area, used the same sampling methodology, and taxonomic identification was conducted by the same team of experts. An increase in the abundance of total cnidaria was found from 1983 to 2011. The siphonophore *Muggiaea atlantica* and the hydromedusa *Aglaura hemistoma* were the most abundant species, while *Muggiaea kochii* presented the largest abundance increment over time. Temperature was the main environmental parameter driving significant differences in the cnidarian community composition, abundance and spatial distribution patterns among the surveys. Our results suggest that in the current climate change scenario, warm-water species abundances will be positively favored, and the community will suffer changes in their latitudinal distribution patterns. We consider it extremely important to study and monitor gelatinous zooplankton in mesoscale spatial areas to understand not only long-term changes in abundances but also changes in their spatial distributions since spatial changes are sensitive indicators of climate change.

## Introduction

There is increasing evidence that ocean warming is driving changes in the abundance, composition and spatial distribution of gelatinous zooplankton worldwide [1,2]. Gelatinous zooplankton are a conspicuous component of planktonic communities, but relatively little is known about their role in marine ecosystems [3,4]. These organisms are highly influenced by oceanographic dynamics, water mass structures, and climate variability [1,5].

Increments in seawater temperature can lead to enhanced abundances of different gelatinous zooplankton groups, such as planktonic cnidarians [6,7]. Following the general global pattern [8], the seawater temperature of the NW Mediterranean has shown an increasing trend over the last few decades [9]. This warming trend was particularly evident in the 1980s and at the end of the 1990s [9,10]. In the Mediterranean Sea, climate change is undoubtedly affecting the basic biology and ecology of organisms as well as the functioning of the pelagic ecosystem (e.g., [11,12]). This scenario in combination with additional impacts (e.g., overfishing, habitat destruction and invasive species) could cause a marine biodiversity loss crisis in the Mediterranean Sea [13].

Analyzing the impact of these events at appropriate spatial scales, temporal scales and biological organization levels, including species and communities, is crucial to accurately predict future changes in marine ecosystems. Monitoring long-term changes in plankton is of great importance because they act as sentinels of changes in marine ecosystems [14]. Luckily, in the Mediterranean Sea, several time series based on one or few sampling stations have been carried out since the late 1960s with high temporal sampling frequencies (weekly, monthly) (see [15]), providing rather extensive knowledge on the gelatinous zooplankton population dynamics and trends in the Mediterranean compared to other areas [16]. These time series allow the identification of long-term changes in the abundance and composition of the planktonic cnidarian community [7,17–19]. Since these series cover a restricted spatial area, in the present work, we raise the need for mesoscale spatial zooplankton studies to complement the existing knowledge of long-term changes in planktonic cnidarians by embracing a large spatial scale. Spatial changes can reflect both species-specific distributional changes and changes in the community distribution patterns. The study and monitoring of these spatial variations in planktonic communities are of significant importance since the plankton act as sensitive indicators of climate change [14,20].

The study of mesoscale spatial areas, in the range of 100 to 1000 km, provides the opportunity to study population and community change rates in relation to variability in physical conditions [21]. In this sense, studies covering a wide grid of stations have revealed changes in distributional patterns of planktonic communities coupled with the distribution of physical phenomena [5,22] and provided valuable knowledge on the ecological role of planktonic cnidarians in some of the most productive marine regions of the world ocean [3,23]. However, under the current climate change scenario, mesoscale changes in planktonic cnidarian distribution patterns have received little attention so far.

Gelatinous zooplankton may benefit from anthropogenic changes such as overfishing, eutrophication, turbidity and hypoxia, among other conditions that can favor jellyfish over fish [24]. In this sense, overfishing is noted as an important factor that enhances gelatinous zooplankton populations by reducing their predators and zooplanktivorous fish competitors [24,25].

This study aims to shed light on the long-term evolution of planktonic cnidarians from a mesoscale spatial point of view. For this purpose, we analyzed (1) species and community spatial distribution patterns, (2) variations in planktonic cnidarian abundance and (3) changes in species richness during three surveys representative of the climatic and anthropogenic changes that have occurred during the last three decades (years: 1983, 2004 and 2011) in the NW Mediterranean. The surveys were carried out during the summer season (June), when high annual abundances of planktonic cnidarians are found [19,26,27].

## Material and methods

**Fig 1:**
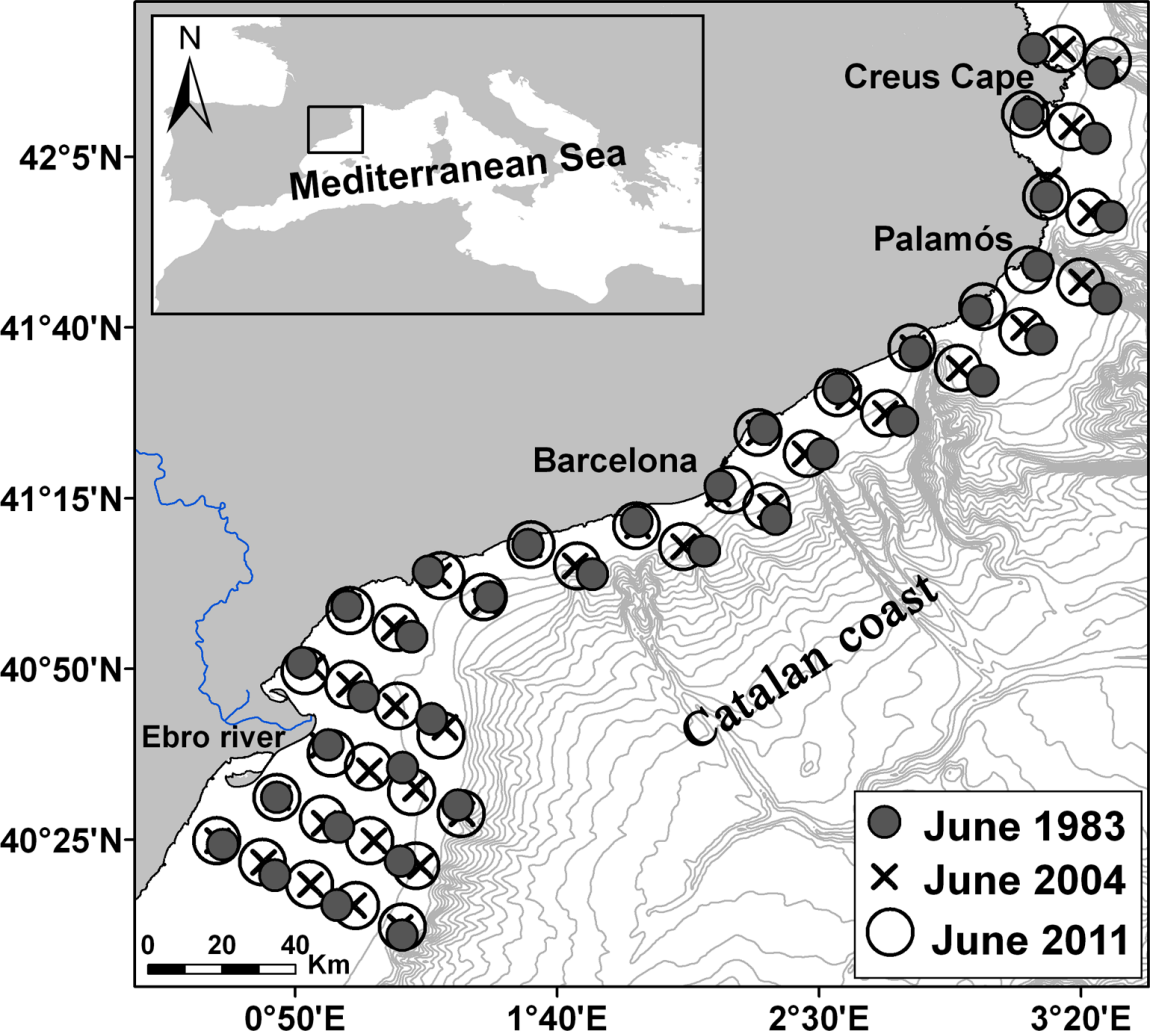
Geographical location of the study area along the Catalan coast in the NW Mediterranean. Grey circles, crosses and open circles represent the sampled stations in the June 1983, 2004 and 2011 surveys, respectively. Grey lines indicate bathymetry (every 100 m).

Three mesoscale surveys were carried out along the Catalan coast (NW Mediterranean) (Fig 1) during June 1983, June 2004 and June 2011 (referred to herein as 1983, 2004 and 2011). In all surveys, the same area was covered (from 40° 13’ N to 42° 22’ N and from 0° 34’E to 3° 25’ E), and the same sampling methodology was applied. Sampling stations were placed along 17 transects perpendicular to the shoreline from near the coast to the shelf break. On each transect, stations were placed between 14 and 16 km apart, and the distance between transects was 18.5 km. The total numbers of sampled stations during the 1983, 2004, and 2011 surveys were 39, 43 and 43, respectively. Vertical profiles of basic hydrographic variables (temperature and salinity) were obtained with a CTD, and water samples were collected at different levels of the water column at each station to determine chlorophyll *a* concentrations (see Sabatés *et al*. [28,29] for methodological descriptions).

Zooplankton was sampled with a Bongo net of 300 μm mesh size. Hauls were oblique from a maximum depth of 200 m to the surface (or from 5 m above the bottom to the surface at stations shallower than 200 m). The volume of filtered water was estimated by a flowmeter placed in the center of the net mouth. Zooplankton samples were fixed immediately after collection in 5% formaldehyde buffered with sodium tetraborate. In the laboratory, planktonic cnidarians (hydromedusae, siphonophores and scyphomedusae) were analyzed at the species level or the lowest possible taxonomical level under a stereomicroscope by the same team of experts. Aliquots were taken only to quantify the most abundant species (e.g., *Muggiaea atlantica* and *Aglaura hemistoma*); for these species, a minimum of 100 individuals were counted in order to calculate the actual number in each sample [30]. Following the method of Pugh [31], nectophores of physonectid siphonophores were counted and divided by 10 to estimate the actual number of colonies sampled. The number of hydromedusae individuals and siphonophore colonies were standardized to number of individuals per 1000 m^3^ of filtered seawater.

In addition, data on monthly sea surface temperatures for the period 1974–2011 were obtained from the L’Estartit Meteorological Station, which is located at the north of the study area (42° 3’N, 3°13’15”E) over a bottom depth of 85 m and operated by Josep Pascual (http://www.meteoestartit.cat). These data were used to assess the long-term evolution of temperature at an annual scale and during the month of June. The mean annual values were plotted against the time-series mean, and the June temperature anomalies were computed as deviations from the June time-series mean.

The permission to conduct the field sampling was given by the Spanish government and sampling points did not include any protected area or private land. We confirm that the studies did not involve endangered or protected species.

### Data analysis

For each species and survey, the mean abundance values, frequency of occurrence (FO, percentage of stations where a taxon occurred) and relative abundance (RA, percentage contribution of a taxon to the total mean abundance of individuals) were calculated. The species richness (S) of the community was estimated as the total number of species found in each survey. Diversity of the whole cnidarian community was calculated using the Shannon diversity index (H’) with a natural logarithm base for each sampled station.

Significant differences in abundance between pairs of years were tested for the total cnidaria abundance and the abundance of the taxa Siphonophorae, Hydromedusae and Scyphomedusae with an analysis of variance using generalized linear models (GLM). The counts of total cnidaria and Siphonophorae, following a Poisson distribution, were analyzed with the function “glm”, and the counts of Hydromedusae and Scyphomedusae, following a binomial negative distribution, were analyzed with the “glm.nb” package [32] and a log link function [33]. The log of the seawater filtered by the net was included as an offset inside all models to eliminate biases due to variable sample sizes [33–35].

To assess whether planktonic cnidarian communities differed among surveys, a non-metric multidimensional scaling ordination (nMDS) of all sampling stations was performed. Species abundances were log (x+1) transformed and an ordination by a Bray-Curtis dissimilarity matrix was performed using the r-language function metaMDS available in the “vegan” package [36]. Subsequently, an adonis permutation multivariate analysis of variance and pairwise tests were used to test for significant differences in the cnidarian communities between surveys. The adonis analysis and pairwise test were performed with the r-language adonis function available in the “vegan” package [36]. To quantify the contribution of the species to the dissimilarity between the pairs of surveys, a similarity percentages routine (SIMPER) was performed.

A canonical correspondence analysis (CCA) was performed in order to identify the environmental factors that most strongly influenced the differences in the planktonic cnidarian communities among surveys. The statistical significance of the axes of the CCA was evaluated using a permutation test with 999 permutations. Additionally, a CCA for each survey was performed to investigate which environmental factor contributed the most to the spatial distribution of the community in each year and explore whether the weight of the environmental factors differed among the years. For both CCA analyses, the collinearity between pairs of environmental variables was evaluated by pairwise scatterplots and Pearson’s correlation coefficients with a cut-off value of |0.5| [33]. The variables chlorophyll *a* and salinity were collinear, and salinity was retained for the analyses because it was previously observed to be one of the most determinant parameters in the distribution of planktonic cnidarians in the area [37,38]. The species matrix used in all the statistical analyses was composed by those species with more than five presences (individuals) and/or present in more than 2 stations during the three surveys. GLM and nMDS statistical analyses were carried out in the free statistical software R, version 3.0.2 [39], and the SIMPER and CCA analyses were performed in the PAST free software [40]. Maps of the horizontal distribution of the environmental parameters, using spline interpolations, species abundance and score values for the first axis from CCAs, were generated by the ArcGIS 10.2 software. The Catalano-Balearic Sea bathymetric chart [41] was used to represent the bathymetry at 100 m intervals.

## Results

### Environmental conditions

**Fig 2:**
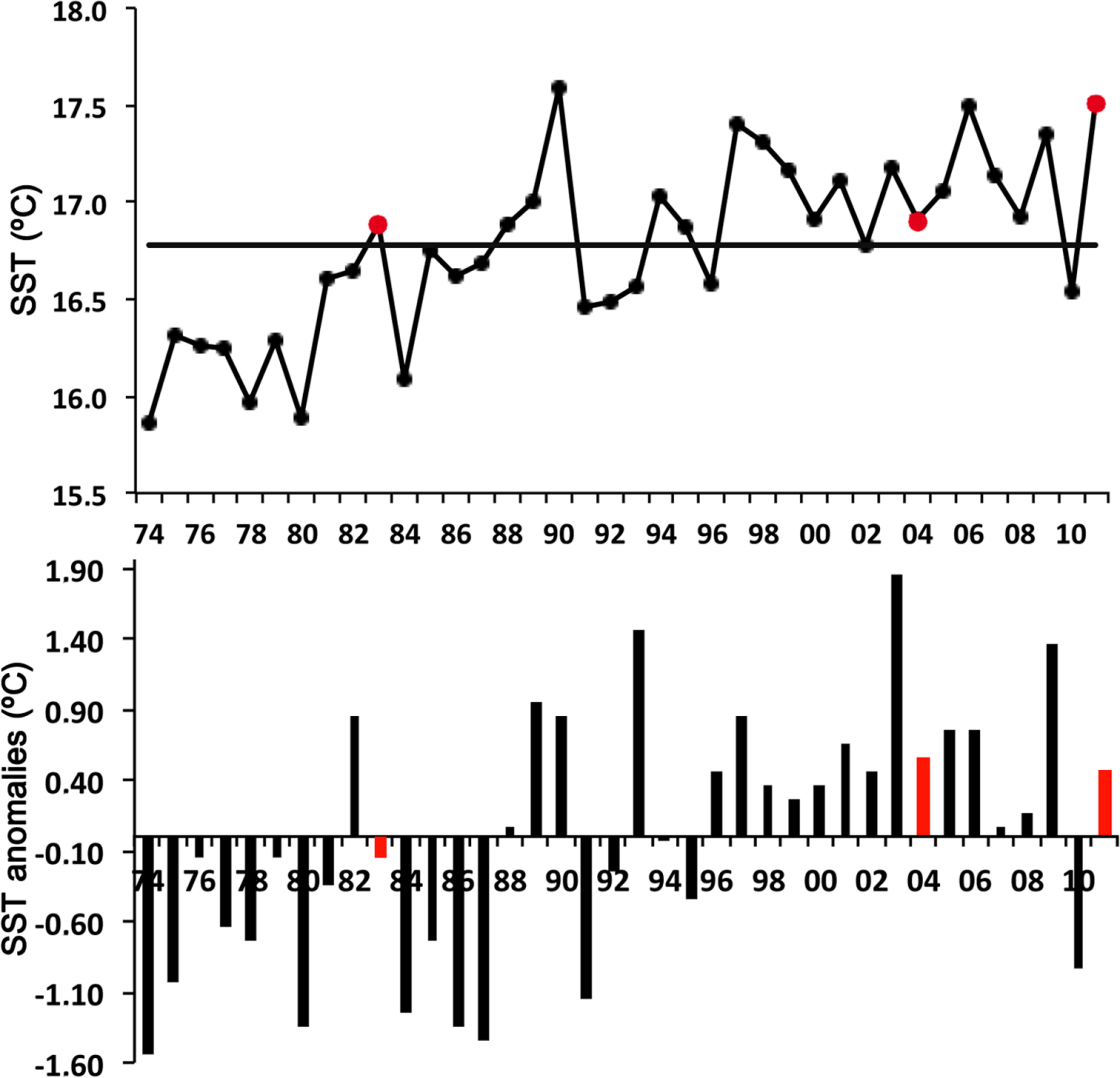
**Upper panel: Annual mean sea surface temperature (SST) over the period 1974–2011.** The horizontal line represents the mean value of the long-term series, and red points represent the studied years (1983, 2004 and 2011). **Lower panel: SST anomalies for the summer months of June over the period 1974–2011.** Red bars represent the studied years. Data from the L’Estartit Meteorological Station.

The long-term evolution of the annual mean temperature from L’Estartit (1974–2010) showed that the 1980s were characterized by values below the mean, while values were mostly above the mean from the late 1990s to the end of the study period (Fig 2). For the June months, the tendency was similar, with 1983 displaying a negative anomaly and 2004 and 2011 displaying positive anomalies, with the highest positive anomaly in 2004 (Fig 2).

**Fig 3:**
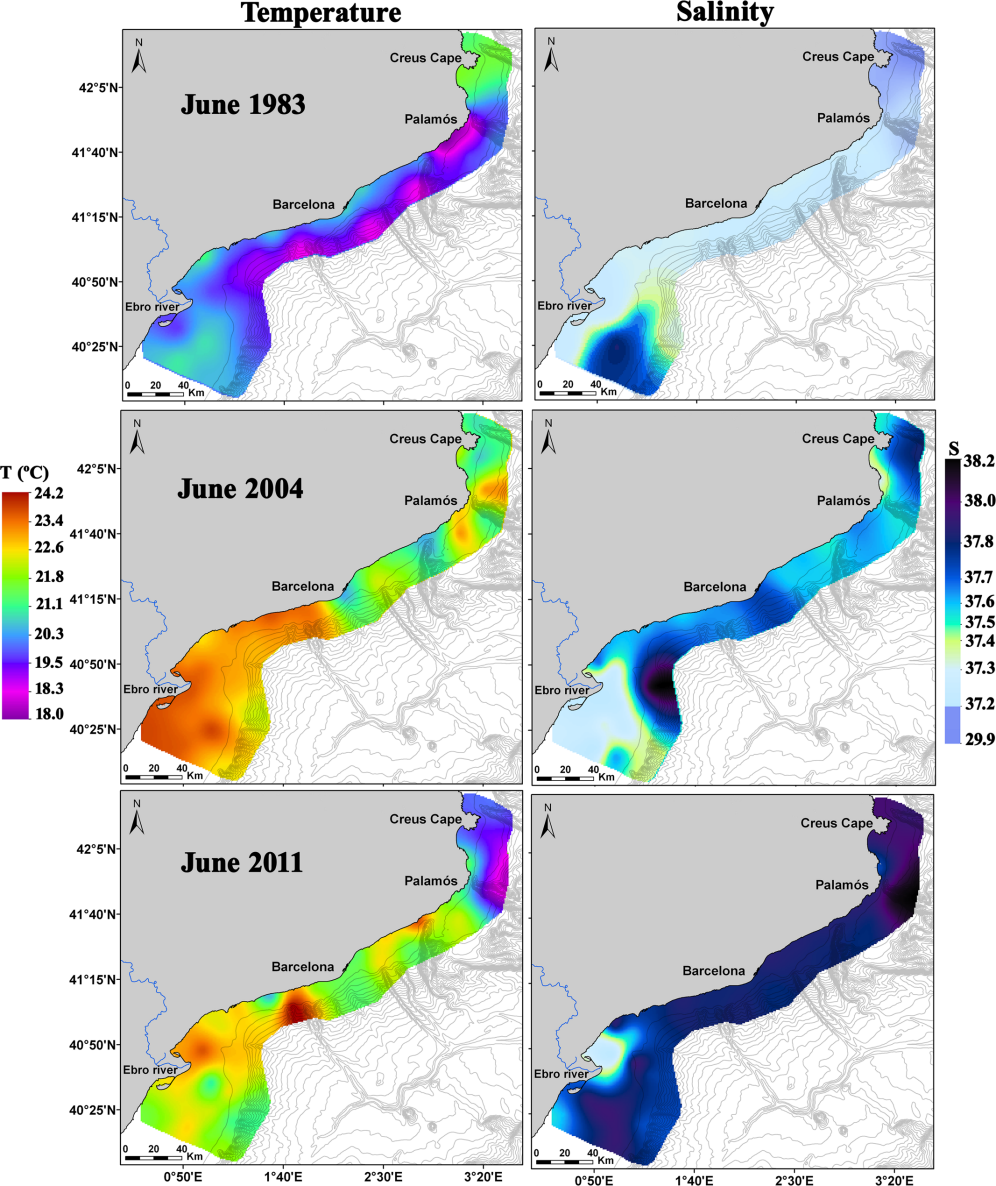
Surface temperature and salinity in June 1983, 2004 and 2011 along the Catalan coast.

Among the three surveys, sea surface temperature showed the lowest values in 1983 (20.1 ± 0.8°C) and the highest in 2004 (22.5 ± 1.1°C), while the intermediate values found during 2011 (21.9 ± 1.1°C) were closer to those of 2004 than those of 1983 (Table 1). In 1983, maximum temperatures were recorded in the northernmost part of the area and near the shore all along the coast (Fig 3). During 2004 and 2011, a marked thermal front was located in the northern half of the study area, separating the cold waters in the north from the warmer waters in the south (Fig 3). In 2004, the main thermal front was located at approximately 42° 00’N and had a temperature difference of nearly 1°C, and a secondary surface temperature front was detected off Barcelona. In June 2011, the main thermal front was observed off Palamós and had a temperature difference of 1° C (Fig 3).

**Table 1:** Values of the environmental parameters along the Catalan coast during the June surveys of 1983, 2004 and 2011.

	June 1983			June 2004			June 2011		
	Mean ± SD	Min.	Max.	Mean ± SD	Min.	Max.	Mean ± SD	Min.	Max.
**Depth (m)**	90 ± 77	15	300	107 ± 99	21	540	108 ± 112	27	685
**T (°C)**	20.13 ± 0.84	18.06	21.77	22.50 ± 1.05	20.48	23.79	21.90 ± 1.09	19.51	24.14
**S**	36.49 ± 1.86	29.85	37.84	37.55 ± 0.20	37.17	38.14	37.78 ± 0.12	37.34	37.99
**Chl *a* (μg l**^**-1**^**)**	0.09 ± 0.09	0.01	0.34	0.23 ± 0.15	0.05	0.80	0.08 ± 0.04	0.03	0.25

Depth = bottom depth, T = sea surface temperature, S = sea surface salinity, Chl *a* = sea surface chlorophyll *a*, SD = standard deviation, Min. = minimum value, Max. = maximum value.

In the three surveys, the most notable feature of the horizontal surface salinity distributions was the presence of low salinity patches (<37.4) in the southern part of the area near the Ebro River mouth (Fig 3). All along the area and near the coast, salinity was generally lower than over the shelf break. In 1983, a surface plume of freshwater coming from the Rhone River was detected in the northernmost part of the area, with salinity values reaching 29.9 (Table 1; Fig 3), while below 20 m, the salinity values were normal for the area and ranged between 37.1 and 38.0 [42].

### Species composition and abundance

**Fig 4:**
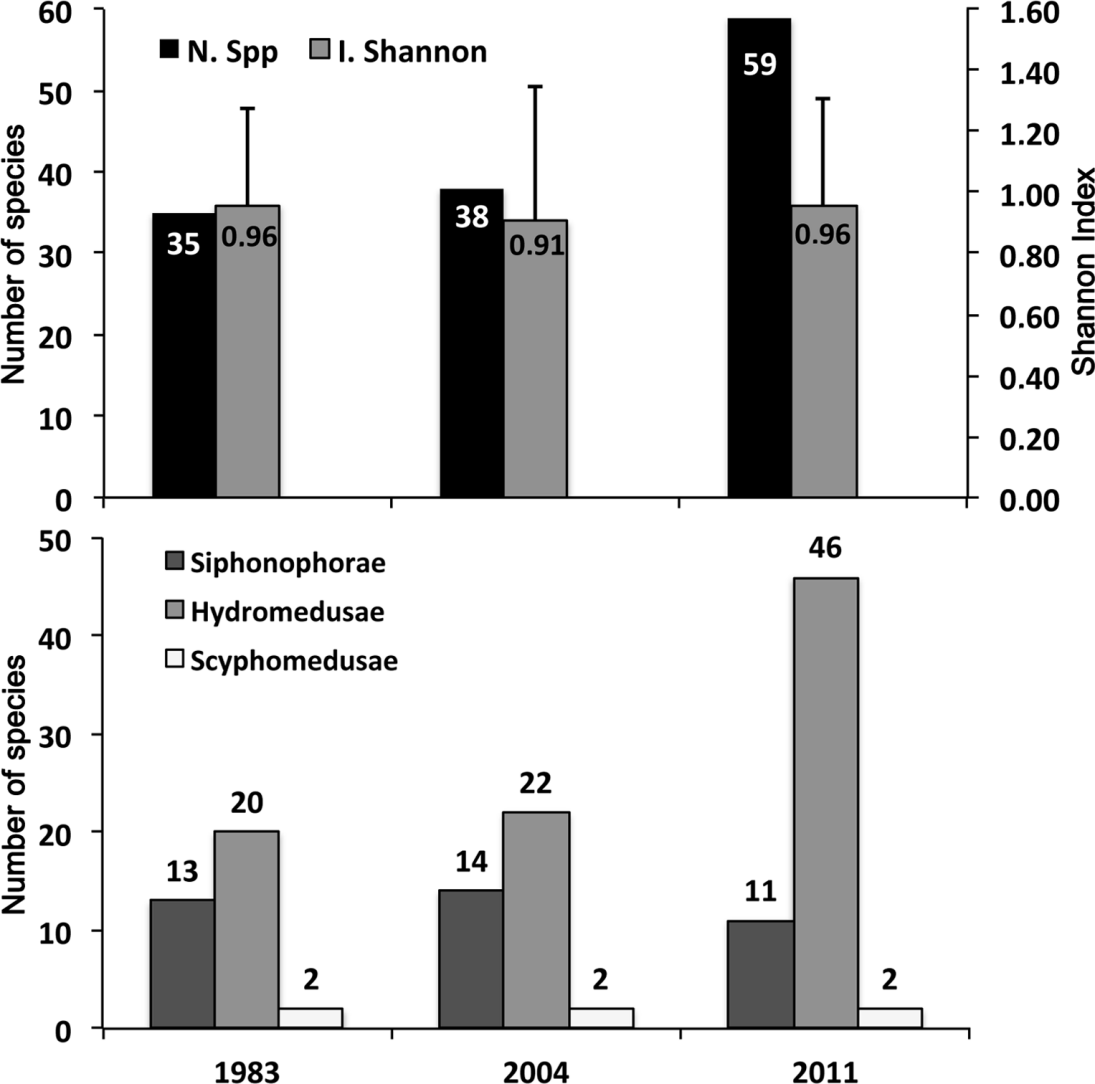
Upper panel: Species richness (total number of species; N. Spp) and diversity (Shannon index in the secondary axis; I. Shannon) for the total cnidarian community in each survey, June 1983, 2004 and 2011. Lower panel: Number of species of the cnidarian taxa Siphonophorae, Hydromedusae and Scyphomedusa in each survey, June 1983, 2004 and 2011.

In the three surveys, a total of 77 species of planktonic cnidarians were found, comprising 19 siphonophores, 55 hydromedusae and 3 scyphomedusae. In 1983 and 2004, the number of species, 35 and 38 respectively, was similar, while the highest number of species, 59, was recorded in 2011 (Table 2; Fig 4). Nonetheless, the Shannon diversity index (H’) remained constant over time: 0.96 ± 0.31 (range: 0.2–1.6) for 1983, 0.91 ± 0.44 (range: 0.11–1.82) for 2004 and 0.96 ± 0.35 (range: 0.12–1.64) for 2011 (Fig 4).

**Table 2:** Mean ± SD abundance values and range (Ind.1000 m^-3^), relative abundance (RA) and frequency of occurrence (FO) for all species found during the June surveys of 1983, 2004 and 2011 along the Catalan coast.

	June 1983				June 2004				June 2011			
	Mean ± SD	Range	RA	FO	Mean ± SD	Range	RA	FO	Mean ± SD	Range	RA	FO
**Total Cnidaria**	**7223.65 ± 6347.65**	**120–21875**	**100**	**100**	**9045.81 ± 5615.91**	**223–21578**	**100**	**100**	**14300.47 ± 12809.86**	**330–59181**	**100**	**100**
**Siphonophorae**	**3962.78 ± 3877.99**	**80–15812**	**54.86**	**100**	**7499.32 ± 4947.52**	**177–19230**	**83.00**	**100**	**8307.98 ± 7750.01**	**255–30511**	**58.10**	**100**
*Abylopsis eschscholtzi*	0.51 ± 3.20	0–20	0.01	2.6	-	-	-	-	0.53 ± 2.42	0–12	0.004	4.7
*Abylopsis tetragona*	68.97 ± 60.08	0–250	0.95	87.2	136.80 ± 152.73	0–582	1.51	90.7	23.42 ± 23.88	0–101	0.16	93.0
*Chelophyes appendiculata*	15.38 ± 21.62	0–90	0.21	53.9	48.85 ± 47.97	0–190	0.54	88.4	121.85 ± 102.73	0–475	0.85	93.0
*Eudoxoides spiralis*	0.77 ± 2.70	0–10	0.01	7.7	0.06 ± 0.37*	0–2	0.001	2.3	-	-	-	-
*Lensia conoidea*	46.92 ± 90.82	0–420	0.65	43.6	46.23 ± 84.16	0–442	0.51	62.8	1.19 ± 4.24	0–23	0.01	9.3
*Lensia fowleri*	-	-	-	-	0.14 ± 0.64	0–3	0.002	4.7	-	-	-	-
*Lensia meteori*	-	-	-	-	0.43 ± 2.85	0–19	0.005	2.33	-	-	-	-
*Lensia subtilis*	11.54 ± 43.32	0–260	0.16	18.0	283.44 ± 239.55	19–1203	3.13	100	16.53 ± 27.42	0–104	0.12	37.2
*Lensia subtiloides*	-	-	-	-	-	-	-	-	4.69 ± 23.01	0–148	0.03	9.3
*Muggiaea atlantica*	3972.56 ± 3928.31	0–15770	54.99	94.9	6867.46 ± 4934.27	52–18722	75.92	100	7836.03 ± 7653.52	60–29758	54.80	100
*Muggiaea kochi*	1.54 ± 5.87	0–30	0.02	7.7	84.62 ± 135.39	0–795	0.94	95.4	225.68 ± 258.77	0–1104	1.58	81.4
*Sulculeolaria chuni*	-	-	-	-	0.18 ± 0.83	0–5	0.002	4.7	0.08 ± 0.53*	0–4	0.001	2.3
*Hippopodius hippopus*	0.77 ± 4.80	0–30	0.01	2.6	-	-	-	-	-	-	-	-
*Agalma elegans*	11.79 ± 36.55	0–190	0.16	18.0	0.15 ± 0.67	0–3	0.002	4.7	-	-	-	-
*Agalma okeni*	2.56 ± 16.01	0–100	0.04	2.6	-	-	-	-	-	-	-	-
*Halistemma rubrum*	32.05 ± 88.83	0–450	0.44	18.0	3.97 ± 5.23	0–27	0.04	55.8	73.32 ± 83.94	0–486	0.51	95.4
*Marrus orthocanna*	1.54 ± 9.61	0–60	0.02	2.6	-	-	-	-	-	-	-	-
*Nanomia bijuga*	-	-	-	-	26.52 ± 39.18	0–226	0.29	90.7	4.68 ± 30.66	0–201	0.03	2.3
*Physophora hydrostatica*	-	-	-	-	0.49 ± 1.32	0–6	0.01	14.0	-	-	-	-
**Hydromedusae**	**2519.49 ± 3075.62**	**0–12193**	**34.88**	**94.9**	**1464.46 ± 1503.41**	**20–5957**	**16.19**	**100**	**5213.09 ± 6249.03**	**15–28670**	**36.45**	**100**
**Order Anthoathecata**	**585.27 ± 1593.62**	**0–7538**	**23.23°**	**61.5**	**22.07 ± 28.48**	**0–150**	**1.51°**	**83.7**	**690.52 ± 1570.49**	**0–8855**	**13.25°**	**83.7**
*Bougainvillia cf*. *muscus*	-	-	-	-	0.12 ± 0.78*	0–5	0.001	2.3	-	-	-	-
*Koellikerina fasciculata*	0.26 ± 1.60*	0–10	0.004	2.6	-	-	-	-	-	-	-	-
*Lizzia blondina*	539.23 ± 1624.49	0–7550	7.46	38.5	0.53 ± 1.90	0–10	0.01	9.3	259.22 ± 712.13	0–3592	1.81	34.9
*Thamnostoma dibalium*	-	-	-	-	-	-	-	-	0.60 ± 3.03	0–19	0.004	4.7
*Eucodonium brownei*	-	-	-	-	-	-	-	-	0.07 ± 0.45*	0–3	0.0005	2.3
*Podocoryna carnea*	1.79 ± 8.54	0–50	0.02	5.1	-	-	-	-	402.20 ± 1443.00	0–8855	2.81	25.6
*Podocorynoides minima*	14.36 ± 72.14	0–440	0.20	7.7	-	-	-	-	7.74 ± 48.01	0–315	0.05	7.0
*Podocorynoides minuta*	50.26 ± 215.29	0–1300	0.70	12.8	-	-	-	-	14.93 ± 91.37	0–599	0.10	4.65
*Hydractinia sp*.	-	-	-	-	-	-	-	-	0.82 ± 3.31	0–17	0.01	7.0
*Amphinema dinema*	0.51 ± 2.23	0–10	0.01	5.1	-	-	-	-	0.24 ± 1.58*	0–10	0.002	2.3
*Amphinema rubrum*	-	-	-	-	0.18 ± 0.88	0–5	0.002	4.7	-	-	-	-
*Amphinema turrida*	-	-	-	-	-	-	-	-	0.10 ± 0.67*	0–4	0.0007	2.3
*Merga tregoubovii*	-	-	-	-	0.26 ± 1.22	0–7	0.003	4.7	0.65 ± 4.28	0–28	0.005	2.3
*Leuckartiara brownei*	-	-	-	-	-	-	-	-	0.16 ± 1.05*	0–7	0.001	2.3
*Leuckartiara nobilis*	-	-	-	-	0.05 ± 0.35*	0–2	0.001	2.3	0.08 ± 0.53*	0–3	0.001	2.3
*Leuckartiara octona*	0.77 ± 2.70	0–10	0.01	7.7	5.08 ± 7.30	0–29	0.06	51.2	0.90 ± 2.02	0–8	0.01	18.6
*Rhathkea octopunctata*	-	-	-	-	-	-	-	-	0.17 ± 1.10*	0–7	0.001	2.3
*Rhabdoon singulare*	-	-	-	-	0.06 ± 0.36	0–2	0.001	2.3	-	-	-	-
*Ectopleura dumortierii*	-	-		-	-	-	-	-	0.17 ± 1.10*	0–7	0.001	2.3
*Hybocodon prolifer*	0.26 ± 1.60*	0–10	0.004	2.6	-	-	-	-	0.17 ± 1.09	0–7	0.001	2.3
*Velella velella* (col.)	-	-	-	-	15.16 ± 31.44	0–148	0.17	62.8	0.09 ± 0.57*	0–4	0.001	2.3
*Zanclea sp*.	-	-	-	-	-	-	-	-	0.16 ± 1.05*	0–7	0.001	2.3
*Zanclea sessilis*	-	-	-	-	-	-	-	-	0.07 ± 0.46*	0–3	0.0005	2.3
*Codonium proliferum*	-	-	-	-	-	-	-	-	0.24 ± 1.57	0–10	0.002	2.3
*Coryne sp*.	-	-	-	-	-	-	-	-	0.18 ± 1.19*	0–8	0.001	2.3
*Corymorpha annulata*	-	-	-	-	-	-	-	-	0.17 ± 1.09	0–7	0.001	2.3
*Corymorpha bigelowi*	-	-	-	-	-	-	-	-	0.07 ± 0.45*	0–3	0.0005	2.3
*Corymorpha forbesii*	-	-	-	-	-	-	-	-	0.41 ± 2.71	0–18	0.003	2.3
*Corymorpha nutants*	-	-	-	-	-	-	-	-	0.07 ± 0.45*	0–3	0.0005	2.3
*Euphysa aurata*	0.26 ± 1.60*	0–10	0.004	2.6	0.62 ± 1.85	0–9	0.01	11.6	0.84 ± 2.34	0–12	0.01	16.3
**Order Leptothecata**	**12.18 ± 26.90**	**0–150**	**0.48°**	**46.2**	**28.42 ± 39.39**	**0–167**	**1.94°**	**79.1**	**149.21 ± 473.68**	**0–3063**	**2.86°**	**86.1**
*Eirene viridula*	2.82 ± 6.86	0–30	0.04	18.0	-	-	-	-	0.23 ± 1.18	0–7	0.002	4.7
*Eutima gegenbauri*	-	-	-	-	0.09 ± 0.62*	0–4	0.001	2.3	0.12 ± 0.78*	0–5	0.001	2.3
*Eutima gracilis*	-	-	-	-	0.24 ± 1.55*	0–10	0.003	2.3	-			
*Helgicirrha cari*	-	-	-	-	-	-	-	-	0.46 ± 2.43	0–16	0.003	4.7
*Helgicirrha schulzii*	0.26 ± 1.60	0–10	0.004	2.6	12.50 ± 20.17	0–87	0.14	58.1	0.54 ± 3.56	0–23	0.004	2.3
*Neotima lucullana*	0.26 ± 1.60*	0–10	0.004	2.6	-	-	-	-	0.10 ± 0.67*	0–4	0.001	2.3
*Guillea* sp.	-	-	-	-	0.13 ± 0.85*	0–6	0.001	2.3	-	-	-	-
*Laodicea undulata*	-	-	-	-	-	-	-	-	0.30 ± 1.94	0–13	0.002	2.3
*Lovenella clausa*	-	-	-	-	-	-	-	-	0.99 ± 5.74	0–37	0.01	4.7
*Earleria sp*.	-	-	-	-	-	-	-	-	0.30 ± 1.51	0–9	0.002	4.7
*Mitrocomella brownei*	-	-	-	-	-	-	-	-	0.67 ± 3.26	0–21	0.005	7.0
*Tiaropsidium mediterraneum*	-	-	-	-	0.11 ± 0.73	0–5	0.001	2.3	0.07 ± 0.46*	0–3	0.0005	2.3
*Clytia hemisphaerica*	2.31 ± 9.86	0–60	0.03	10.3	-	-	-	-	5.24± 7.83	0–37	0.04	46.5
*Clytia spp*.	-	-	-	-	3.44 ± 6.69	0–31	0,04	34.9	0.47 ± 3.07	0–20	0.003	2.3
*Obelia spp*.	7.44 ± 25.62	0–150	0.10	18.0	11.91 ± 22.36	0–107	0.13	53.5	139.71 ± 472.65	0–3063	0.98	58.1
**Order Narcomedusae**	**3.22 ± 10.99**	**0–66**	**0.13°**	**20.5**	**22.25 ± 31.06**	**0–130**	**1,52°**	**72.1**	**23.27 ± 32.01**	**0–135**	**0.45°**	**67.4**
*Cunina sp*.	-	-	-	-	-	-	-	-	0.12 ± 0.78*	0–5	0.001	2.3
*Solmissus albescens*	0.26 ± 1.60*	0–10	0.004	2.6	-	-	-	-	2.28 ± 9.05	0–52	0.02	9.3
*Solmaris flavescens*	0.26 ± 1.60*	0–10	0.004	2.6	-	-	-	-	-	-	-	-
*Solmaris solmaris*	-	-	-	-	-	-	-	-	0.10 ± 0.68*	0–4	0.0007	2.3
*Solmundella bitentaculata*	3.08 ± 11.51	0–70	0.04	15.4	22.25 ± 31.06	0–130	0.25	72.1	22.85 ± 32.22	0–135	0.16	65.1
**Order Trachymedusae**	**1918.81 ± 2247.85**	**0–10723**	**76.16°**	**92.3**	**1391.72 ± 1469.64**	**20–5724**	**95.03°**	**100**	**4350.09 ± 5901.97**	**7–28459**	**83.5°**	**100**
*Liriope tetraphylla*	-	-	-	-	2.86 ± 5.83	0–31	0.03	34.9	-	-	-	-
*Aglaura hemistoma*	1893.33 ± 2240.77	0–10690	26.21	92.3	1159.16 ± 1445.41	0–5605	12.81	97.7	4341.79 ± 5898.75	4–28448	30.36	100
*Persa incolorata*	37.95 ± 127.34	0–730	0.53	18.0	105.32 ± 372.36	0–2058	1.16	65.1	0.52 ± 2.09	0–13	0.004	9.3
*Rhopalonema funerarium*	-	-	-	-	4.87 ± 20.60	0–133	0.05	20.9	-	-	-	-
*Rhopalonema velatum*	3.33 ± 7.37	0–40	0.05	25.6	119.51 ± 99.96	0–400	1.32	90.7	7.78 ± 8.08	0–29	0.05	67.4
**Scyphomedusae**	**741.39 ± 2030.57**	**0–8961**	**10.26**	**43.6**	**82.03 ± 257.87**	**0–1340**	**0.91**	**53.5**	**779.40 ± 2975.35**	**0–18783**	**5.45**	**69.8**
*Atolla sp*.	-	-	-	-	4.22 ± 7.94	0–42	0.05	34.9	-	-	-	-
*Discomedusa lobata*	0.26 ± 1.60*	0–10	0.004	2.6	-	-	-	-	0.07 ± 0.45*	0–3	0.0005	2.3
*Pelagia noctiluca*	745.13 ± 2041.28	0–8970	10.32	43.6	77.81 ± 255.78	0–1327	0.86	41.9	779.33 ± 2975.37	0–18783	5.45	69.8

*Only 1 individual found

°RA relative to the Hydromedusae group.

**Fig 5:**
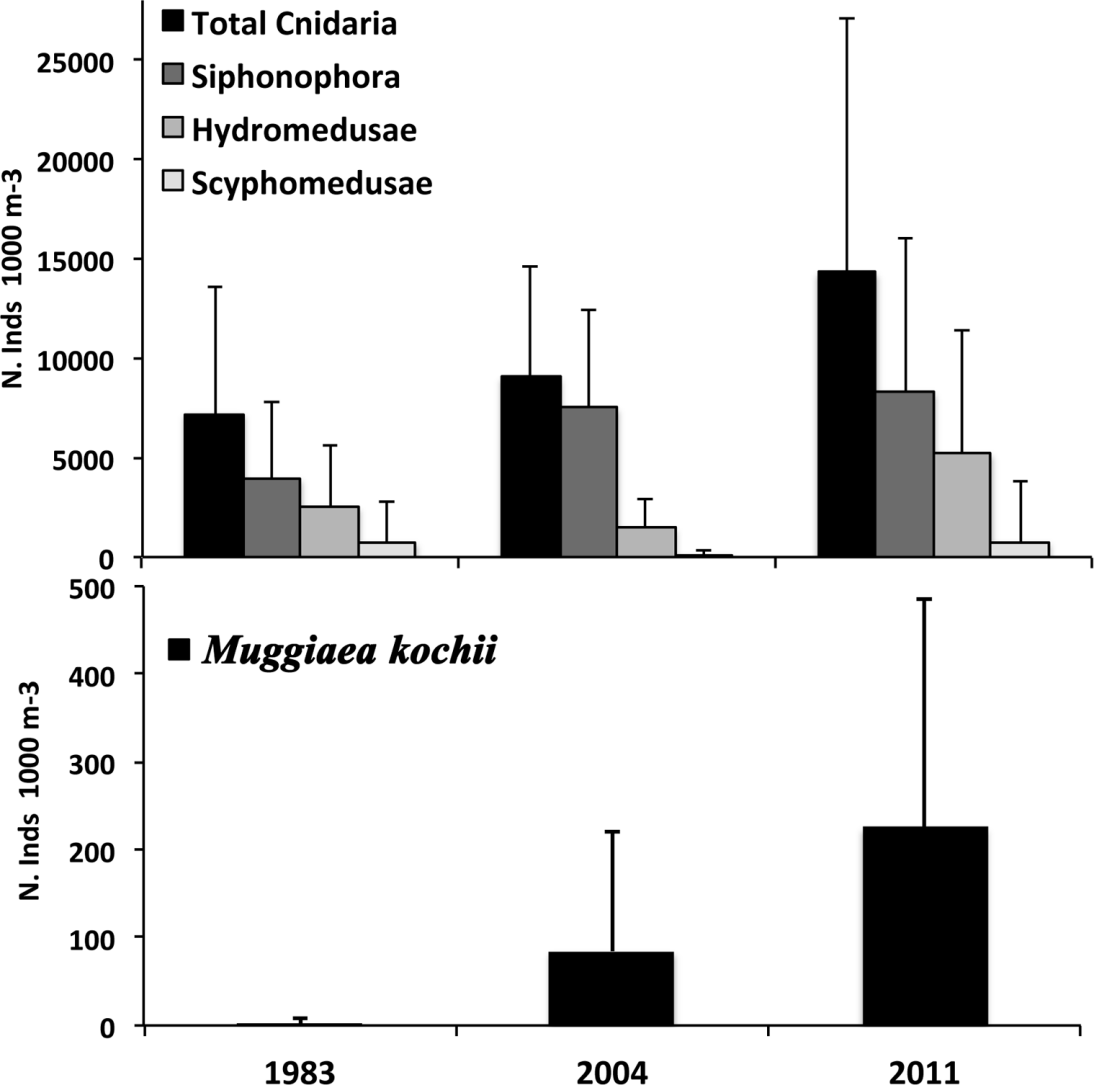
Upper panel: Mean abundance (N. Inds 1000 m^-3^) (+ SD) for total cnidaria, and the taxa Siphonophorae, Hydromedusae and Scyphomedusae in each survey, June 1983, 2004 and 2011. Lower panel: mean abundance (N. Inds 1000 m^-3^) (+ SD) of the calycophoran siphonophore *Muggiaea kochii* in each survey, June 1983, 2004 and 2011.

An increase in the abundance of total cnidaria was found from 1983 to 2011 (Fig 5; Table 3). This tendency was observed for the siphonophorae and hydromedusae taxa but not for the scyphomedusae, the abundance of which did not vary over time (Fig 5; Table 3). The siphonophore *M*. *atlantica* and the hydromedusa *A*. *hemistoma* were the most abundant species during the three surveys (Table 2), and they together accounted for more than 80% of the total cnidarian abundance (Table 2). The calycophoran siphonophore *M*. *kochii* presented the most important abundance increase over time; it was barely found in 1983, and its abundance showed a fortyfold increase in 2004 and a further threefold increase in 2011. In addition, it was widely distributed over the studied area during the last two surveys (Table 2). The species composition of siphonophores and scyphomedusae remained similar over time. However, the hydromedusae taxon showed major changes in its species composition due to the differences in the represented species of the orders Anthoathecata and Leptothecata. The lowest number of species in these two orders occurred during 1983 and 2004. However, more species of these orders were shared between 1983 and 2011 than between 1983 and 2004 (Table 2).

**Table 3:** Results of the analyses of variance using generalized linear models (GLM) for the abundance of total cnidaria, Siphonophorae, Hydromedusae and Scyphomedusae between pairs of years.

	1983–2004		2004–2011		1983–2011	
	z-value	p-value	z-value	p-value	z-value	p-value
**Total Cnidaria**	47.8	<0.001	87.6	<0.001	106.6	<0.001
**Siphonophorae**	72.5	<0.001	22.5	<0.001	86.6	<0.001
**Hydromedusae**	-2.1	<0.05	4.9	<0.001	2.5	= 0.01
**Scyphomedusae**	-3.3	<0.001	3.9	<0.001	0.08	n.s.

### Differences among years

**Fig 6:**
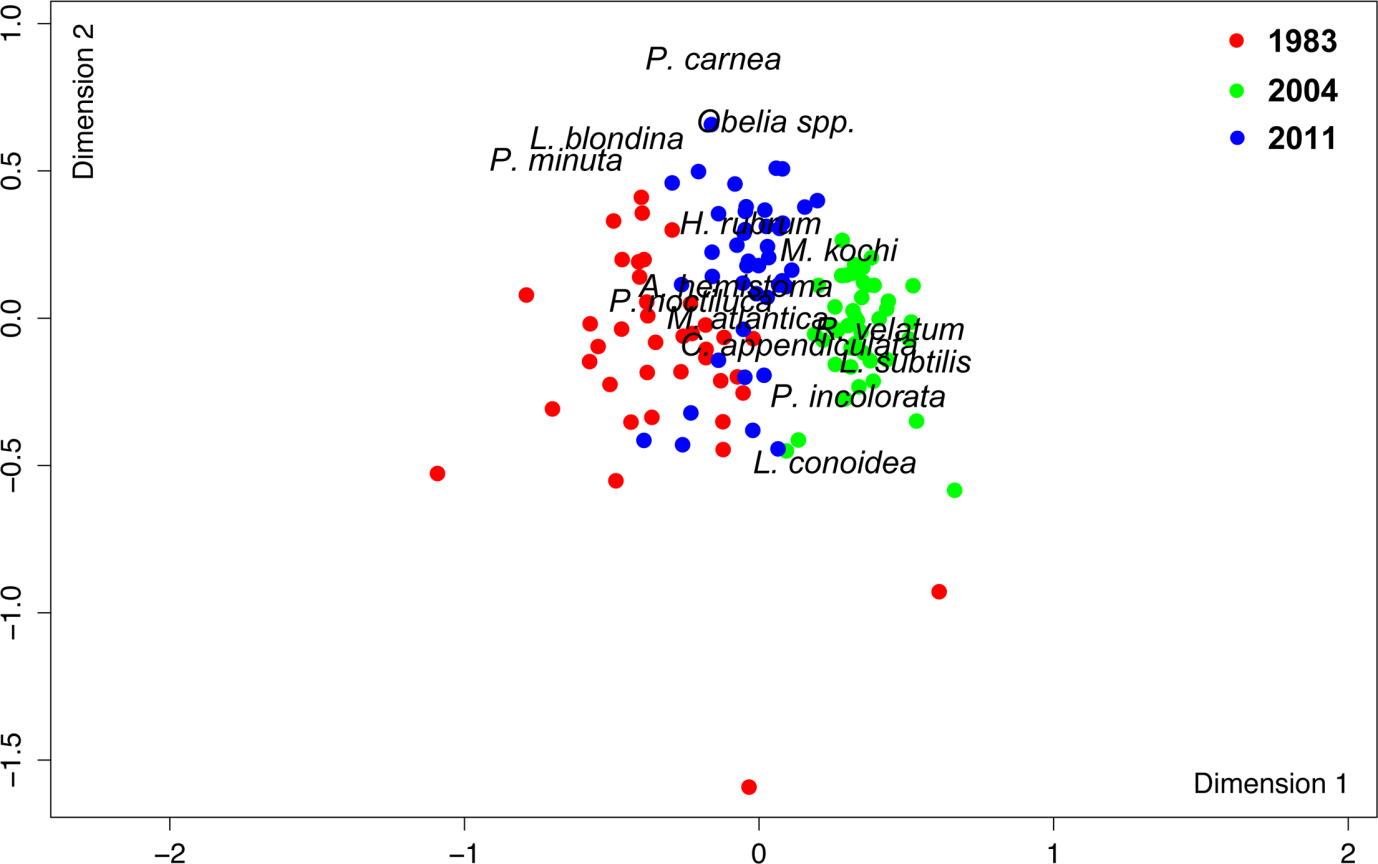
Non-metric multidimensional scaling (nMDS) ordination plot. Species abundances were log (x+1) transformed and an ordination was performed with the Bray-Curtis dissimilarity matrix. Red, green and blue dots symbolize sample stations from June 1983, 2004 and 2011, respectively. A stress estimate of 0.21 was obtained. The most abundant and representative species for each survey are indicated in the plot.

**Fig 7:**
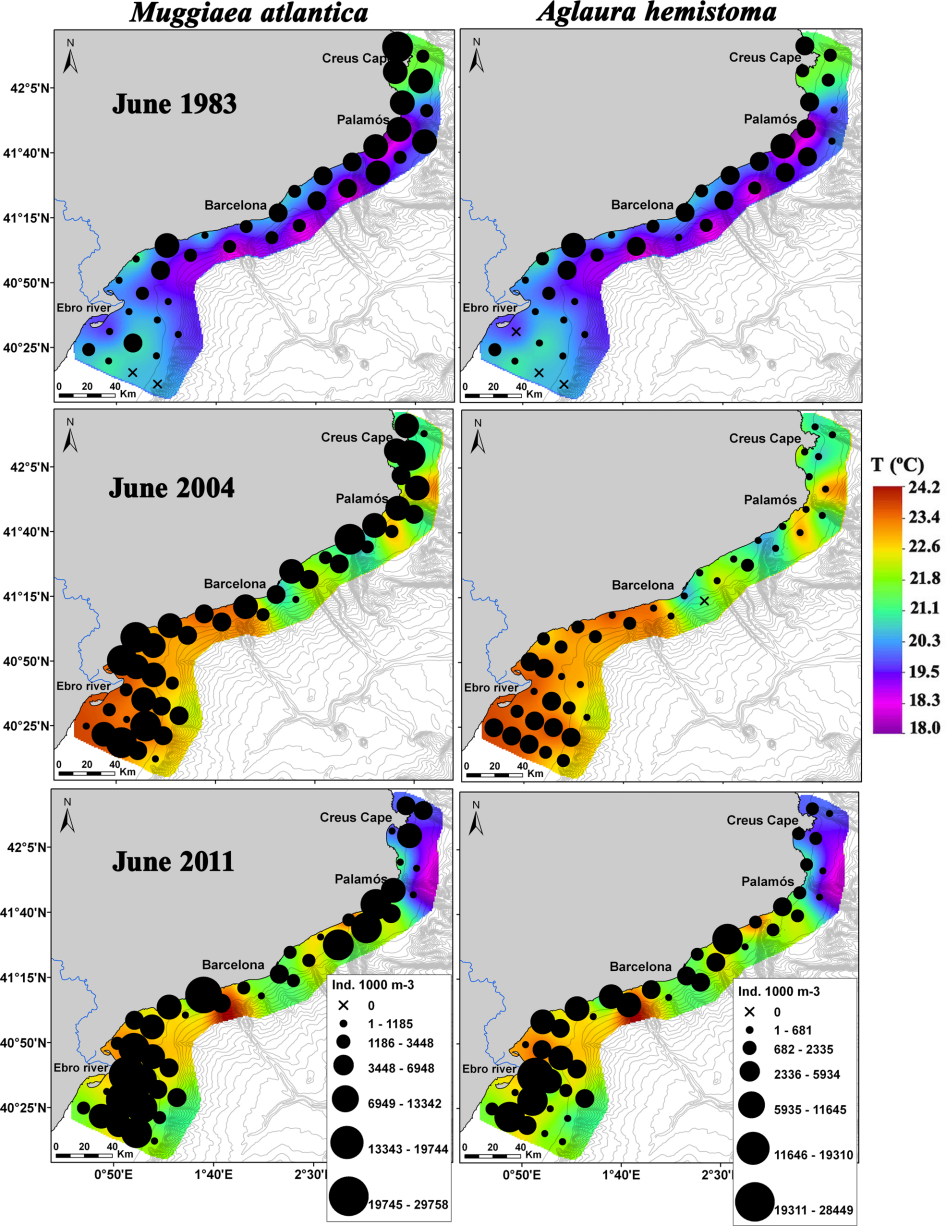
Spatial distribution of the dominant species *Muggiaea atlantica* and *Aglaura hemistoma* overlaid on the surface temperature for each survey. From top to bottom: June 1983, 2004 and 2011.

The nMDS analysis showed that samples from each survey were grouped separately (Fig 6). The 1983 and 2011 stations were located close to each other on the negative side of the first dimension, while the 2004 stations were mostly located on the positive side (Fig 6). The species composition in 1983 was mainly characterized by *Lizzia blondina* and *Podocorynoides minuta*; in 2004, it was characterized by *Lensia subtilis*, *Rhopalonema velatum* and *Persa incolorata*, and in 2011, it was characterized by *Podocoryna carnea*, *Obelia* spp. and *Halistemma rubrum* (Fig 6). Other species such as *Pelagia noctiluca* were mainly found in 1983 and 2011, while *M*. *atlantica* and *A*. *hemistoma* were common in all three of the surveys (Figs 6 and 7; Table 2). The adonis permutation multivariate analysis of variance and subsequent pairwise tests revealed that the communities identified during each cruise were significantly different (p < 0.001) from each other.

**Fig 8:**
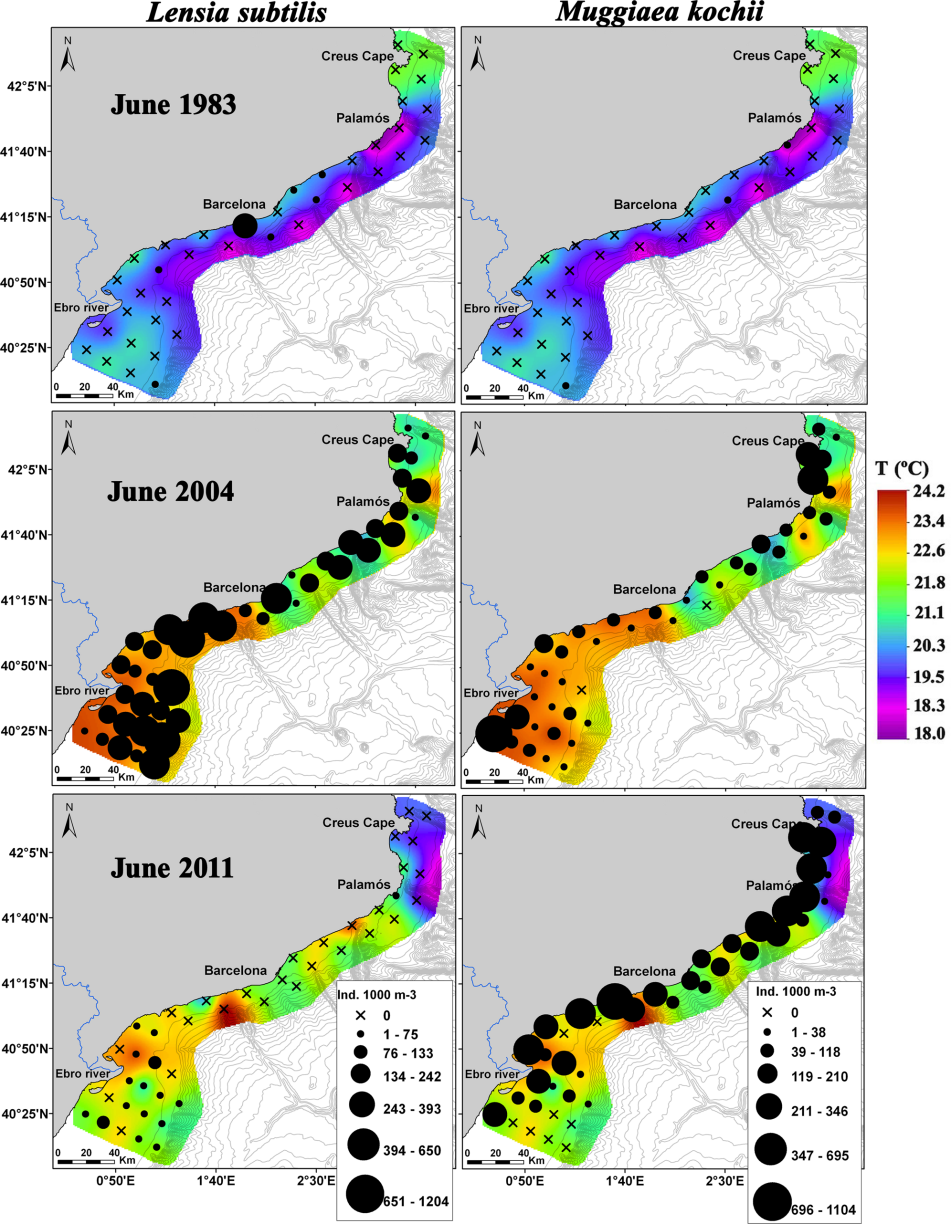
Spatial distribution of the species *Lensia subtilis* and *Muggiaea kochii* overlaid on the surface temperature for each survey. From top to bottom: June 1983, 2004 and 2011.

The SIMPER analysis showed the highest average dissimilarity in species composition between 1983 and 2004 (Table 4). The number of species contributing up to 90% of the dissimilarity ranged between 15 and 20 (Table 4). The three species that had the greatest effect on the dissimilarity between 1983 and 2004 were *L*. *subtilis*, *R*. *velatum* and *M*. *kochii*. Based on their abundances and spatial distributions, these three species were much more abundant and widely spread during 2004 than during 1983 (Table 2; Fig 8). *Lensia subtilis* (Fig 8) and *R*. *velatum* (not shown) had similar spatial distributions in the three surveys. The species that contributed the most to the dissimilarities between 2004 and 2011 were *L*. *subtilis*, *R*. *velatum*, and *P*. *noctiluca*. The first two were more abundant and widespread during 2004 than during 2011, while *P*. *noctiluca* was much more abundant during 2011 (Table 2; Fig 8). Dissimilarities between the first and the last survey were derived mainly from *M*. *kochii*, the physonectid *H*. *rubrum* and *P*. *noctiluca*. The first two species were more abundant and widespread in 2011 (Table 2; Fig 8, only *M*. *kochii* shown). The scyphomedusa *P*. *noctiluca* presented similar abundance values in both years (Table 2); however, it was more widespread in 2011. Furthermore, in 2011, its highest densities were over the shelf and the shelf edge, which contrasts with 1983 when this species was observed close to the coast.

**Table 4:** Species contributions as average dissimilarities (Av. Dis.) to the overall average dissimilarity between each pair of years, as indicated by the SIMPER analysis. Species contributing to the first 90% of the dissimilarity are shown in decreasing order of percentage contribution. Species contributing to the three highest average dissimilarities (one year vs other) are in **bold**.

1983–2004				2004–2011				1983–2011			
Overall average dissimilarity: 54%					46%				49%		
Taxon	Av. Dis.	Contrib. %	Cum. %	Taxon	Av. Dis.	Contrib. %	Cum. %	Taxon	Av. Dis.	Contrib. %	Cum %
***L*. *subtilis***	5.591	**10.34**	10.34	***L*. *subtilis***	3.995	**8.681**	8.681	***M kochi***	5.029	**10.24**	10.24
***R*. *velatum***	4.178	**7.727**	18.07	***R*. *velatum***	2.852	**6.199**	14.88	***H*. *rubrum***	4.335	**8.829**	19.07
***M*. *kochi***	4.157	**7.689**	25.76	***P*. *noctiluca***	2.82	**6.129**	21.01	***P*. *noctiluca***	4.236	**8.628**	27.7
*P*. *noctiluca*	3.078	5.693	31.45	*H*. *rubrum*	2.695	5.857	26.87	*C*. *appendiculata*	3.671	7.477	35.18
*N*. *bijuga*	3.011	5.569	37.02	*A*. *hemistoma*	2.564	5.572	32.44	*L*. *blondina*	3.505	7.139	42.32
*A*. *hemistoma*	2.926	5.411	42.43	*N*. *bijuga*	2.558	5.56	38	*A*. *hemistoma*	3.299	6.72	49.04
*P*. *incolorata*	2.771	5.124	47.55	*Obelia spp*.	2.234	4.855	42.85	*Obelia spp*.	2.885	5.876	54.91
*L*. *conoidea*	2.752	5.09	52.64	*L*. *conoidea*	2.214	4.812	47.67	*M*. *atlantica*	2.703	5.505	60.42
*C*. *appendiculata*	2.564	4.742	57.38	*M*. *kochi*	2.197	4.774	52.44	*S*. *bitentaculata*	2.471	5.033	65.45
*L*. *blondina*	2.397	4.434	61.82	*P*. *incolorata*	2.166	4.707	57.15	*L*. *conoidea*	2.36	4.807	70.26
*A*. *tetragona*	2.378	4.399	66.22	*A*. *tetragona*	2.022	4.395	61.54	*A*. *tetragona*	2.321	4.727	74.98
*S*. *bitentaculata*	2.278	4.212	70.43	*S*. *bitentaculata*	1.858	4.037	65.58	*L*. *subtilis*	2.032	4.138	79.12
*M*. *atlantica*	2.226	4.118	74.55	*C*. *appendiculata*	1.742	3.786	69.37	*R*. *velatum*	1.918	3.907	83.03
*V*. *velella*	1.881	3.479	78.03	*L*. *blondina*	1.738	3.777	73.14	*P*. *carnea*	1.718	3.5	86.53
*H*. *rubrum*	1.779	3.29	81.32	*V*. *velella*	1.59	3.456	76.6	*C*. *hemisphaerica*	1.527	3.11	89.64
*Obelia spp*.	1.742	3.221	84.54	*M*. *atlantica*	1.573	3.42	80.02				
*H*. *schulzei*	1.704	3.152	87.69	*H*. *schulzei*	1.464	3.181	83.2				
*L*. *octona*	1.342	2.482	90.17	*P*. *carnea*	1.236	2.686	85.89				
				*L*. *octona*	1.118	2.429	88.32				
				*C*. *hemisphaerica*	1.094	2.377	90.69				

### Relationships between the community and environmental factors

**Fig 9:**
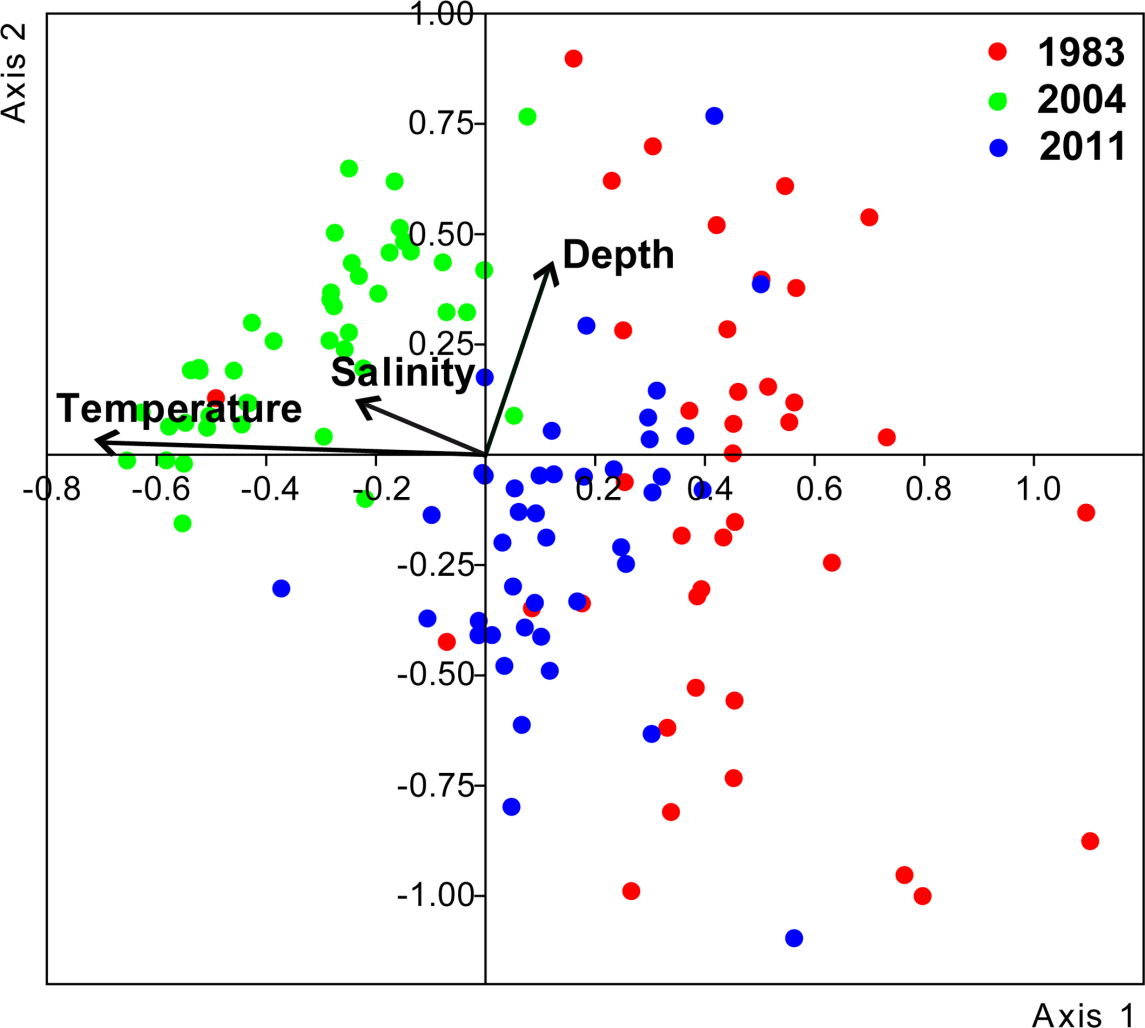
Ordination plot of the canonical correspondence analysis (CCA) showing the relationships between the sampling stations (red for 1983, green for 2004 and blue for 2011) and the environmental variables (arrows).

In the CCA analysis combining all surveys, the first ordination axis was strongly and negatively correlated with temperature (-0.70) and accounted for 63% of the constrained variance (Table 5). Stations from 2004 were grouped on the negative side of the axis, showing their correlation with higher temperatures. The 1983 and 2011 stations were on the positive side of the axis, with those of 1983 especially related to the coldest temperatures (Fig 9). The second ordination axis was positively correlated with depth (0.42), which accounted for 37% of the constrained variance (Table 5). Samples from 1983 and 2011 were spread over both sides of the axis, while those from 2004 were grouped mostly on the positive side, less influenced by the bathymetry (Fig 9). Salinity was barely related to the first axis (-0.22) (Table 5). The permutation test indicated the high significance (p < 0.001) of the two first ordination axes. These results show that, among the measured variables, temperature was the main environmental factor influencing the differences in community composition and abundance among the years.

**Table 5:** Summary of the relationships between the environmental variables and axis 1 from the canonical correspondence analyses (CCA) performed for all surveys combined and for each June survey (1983, 2004 and 2011). The most significant factor in each case is in **bold**. Cons. var. explained = constrained variance explained.

	Axis 1			
	CCA combined	CCA 1983	CCA 2004	CCA 2011
Cons. var. explained	63%	63%	76%	78%
Depth	0.11	**-0.61**	0.56	0.46
Temperature	**-0.70**	0.49	**-0.67**	**-0.55**
Salinity	-0.22	-0.34	0.55	0.29

**Fig 10:**
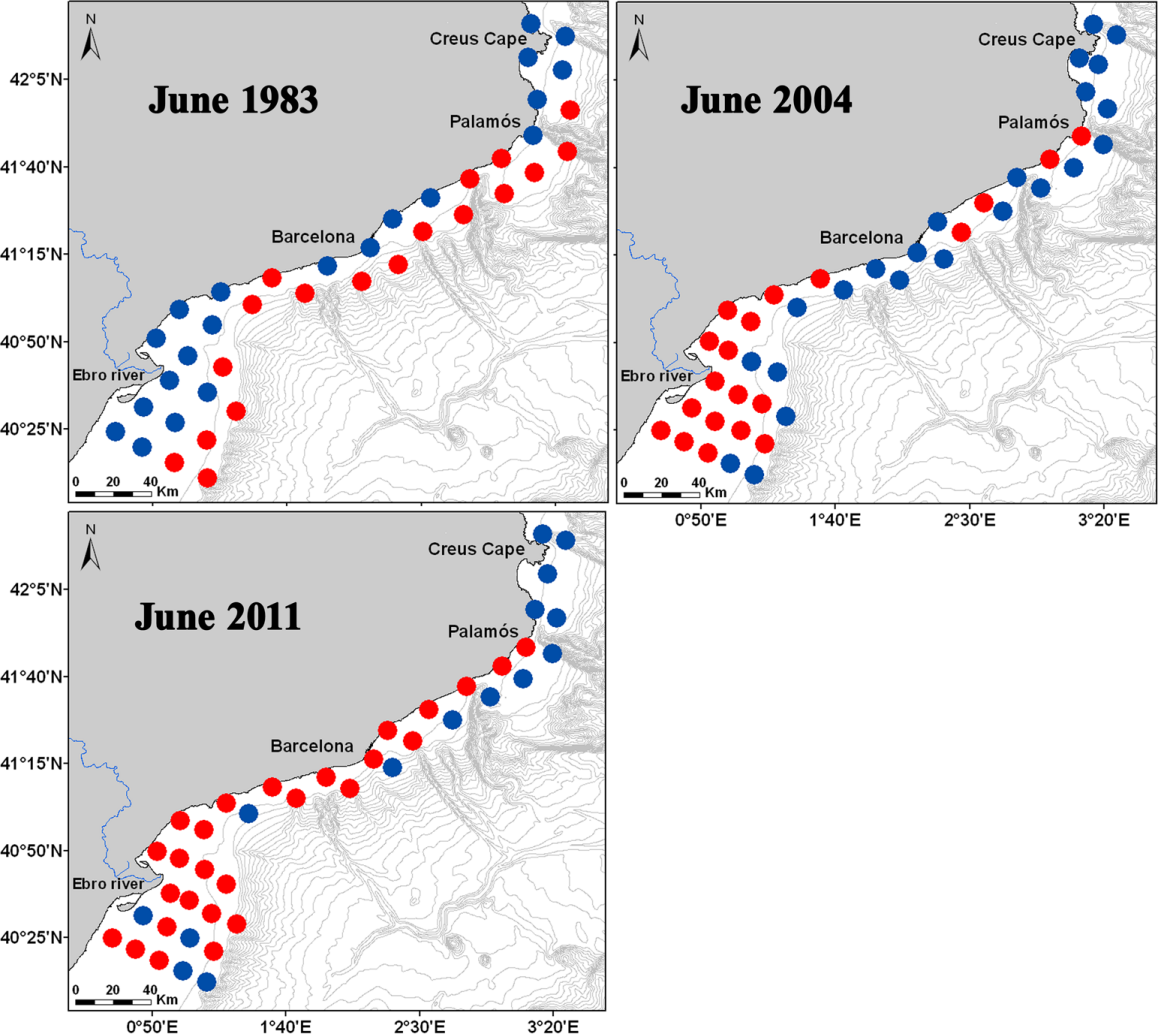
Spatial distributions of the positive and negative score values of each sampled station for the first axis that resulted from the CCA analyses for each survey. Blue dots represent negative score values, and red dots represent positive values.

The results of the CCAs performed for each survey demonstrated that the weight of the main environmental factor (the one highly correlated with the first ordination axis) affecting the spatial distribution of the community differed among the years. In 1983, depth was the main environmental factor, while in 2004 and 2011 the main factor was temperature (Table 5). In addition, a decreasing influence of the depth factor was observed over time (Table 5). In all cases, axis 1 was revealed as significant by the permutation test. The spatial distribution of the positive and negative score values of each sampled station for the first axis clearly shows that the community in 1983 was ordinated in relation to the coast-offshore axis. In contrast, during 2004 and 2011 this pattern was much less clear, and a north-south ordination was noticeable, especially in 2004 (Fig 10).

## Discussion

In the present study, the spatio-temporal variability in the planktonic cnidarian community was investigated during three summer (June) surveys conducted during the last three decades (1983, 2004 and 2011) over a mesoscale spatial area along the Catalan coast. The results showed changes in the community composition as well as an increment in planktonic cnidarian abundances over time. Among the different environmental variables considered, water temperature contributed the most to these changes (Fig 9). Temperature has been suggested as a key factor driving long-term changes in zooplankton communities in several areas worldwide [43,44]. Over the last few decades, seawater temperature in the NW Mediterranean has shown an increasing trend [9], which follows the general global pattern [8]. This warming trend has been particularly evident since the 1980s and at the end of the 1990s [9,10]. The temperature increase along the Catalan coast over the last three decades is well documented [12]. As observed in the long-term evolution of seawater temperature in the present study, Sabatés *et al*. [45] identified two marked temperature shifts; the first one was in the early 1980s, and the second was approximately in 1997.

In the western Mediterranean, long-term series conducted at historic stations (e.g., Villefranche, Naples) over the last three decades have shown that increases in water temperature are associated with changes in the abundance of planktonic cnidarians [7,15,17–19]. Molinero *et al*. [7,17] (studied period: 1966–1993), while studying certain target species (2 siphonophores and 3 hydromedusae), showed a rise in jellyfish abundance related to increments in water temperature during the 1980s and early 1990s. Nevertheless, Licandro *et al*. [19] and García-Comas *et al*. [18] (studied periods; 1974–1999 and 1974–2003, respectively) found that the Siphonophorae and Medusae community stocks did not increase since the late 1980s, despite an increase in sea warming. In our study, which considered a large mesoscale spatial area, the total abundance of planktonic cnidarians increased significantly and progressively over time (Fig 5 and Table 3), and water temperature was the main environmental factor, among the studied parameters, associated with these changes (Fig 9 and Table 5). Salinity gradients have been associated with changes in planktonic cnidarian abundances and community compositions [19,24]. However, in our study, this does not seem to be the case, since this factor barely affected abundance and community composition values (Fig 9 and Table 5) despite the observed surface salinity differences among the surveys (Fig 3 and Table 1).

Studies carried out in the NW Mediterranean from late 1960s reported the dominance of the calycophoran siphonophore *M*. *kochii* until the early 1980s, after which this species abundance abruptly declined [19,46]. We observed a marked and progressive increase in the abundance of this species throughout the study period, and it became widely distributed over the study area in the last two surveys (Table 2; Fig 8). An increase in the abundance of *M*. *kochii* has also been reported in recent years in a estuarine ecosystem in the northeastern Atlantic Ocean [47]. This siphonophore is considered a warm-temperate species [48] and may therefore benefit from the observed increase in water temperature over time (Fig 2). In different areas of the world, an alternate pattern in the abundance of *M*. *kochii* and its congeneric *M*. *atlantica* has been observed [49–51]. Surprisingly, in the present study, the abundance of both species increased simultaneously (Table 2; Figs 7 and 8). However, if we calculate the abundance ratio *M*. *atlantica*:*M*. *kochii*, it decreased over time. The ratio values were: 2000 for June 1983, 80 for June 2004 and 35 for June 2011, indicating that even though the summer abundance of both species increased over time, *M*. *kochii* experienced a higher rate of increase than *M*. *atlantica*. This suggests that, under the current climate change scenario, the warm-temperate species *M*. *kochii* would be favored more than the cold-temperate species *M*. *atlantica*. The abundance ratio between these two species could be used as a proxy for measuring gelatinous zooplankton responses to sea temperature changes. Attention should be paid to the abundance trends and rates of these two congeneric species in different temperate places worldwide as an indicator of ocean warming.

The planktonic cnidarian communities identified during each June survey significantly differed from one another and were clearly segregated by temperature (Figs 6 and 9; Table 5). The siphonophore *L*. *subtilis* and the hydromedusae *R*. *velatum*, both considered warm-water species and abundant during the summer [37,48,52], characterized the community in 2004 (Fig 6). This agrees with the fact that 2004 presented the warmest temperatures (Fig 3; Table 1) and the highest positive anomalies of the three studied periods (Fig 2). The abundance of these two species has been observed to increase under exceptional warm summer conditions in the area [53]. The community in 1983 was influenced by the coldest temperatures of the studied periods, and it was influenced by intermediate temperatures in 2011. The communities in both 1983 and 2011 were segregated by depth (Fig 9). The tiny coastal hydromedusae *L*. *blondina* and *P*. *minuta* [54] characterized the community in 1983 (Fig 6). Both species appear in early spring when seawater is still cold, before the highest yearly temperatures occur [37,55]. The community in 2011 was defined by other small and coastal hydromedusae species, *P*. *carnea* and *Obelia* spp. [56]. *Podocoryna carnea* is found from spring to autumn in the Mediterranean, while the species belonging to the genera *Obelia* have different seasonal peaks and are mostly present throughout all the year [37,57]. Although the aforementioned species were among the most abundant during each corresponding survey (Table 2), the siphonophore *M*. *atlantica* and the hydromedusae *A*. *hemistoma* were the dominant species during all surveys (Table 2), suggesting they can tolerate wide environmental ranges and are able to exploit favorable conditions more efficiently than other species [19]. The dominance of these two species in the NW Mediterranean is a phenomenon that has been observed since the early 1980s, when *M*. *atlantica* and *A*. *hemistoma* outcompeted the previously dominant species, *M*. *kochii* and *L*. *blondina* [19,37,46].

Although no significant differences in species diversity (H’) were found among the three surveys, in 2011, the species richness was considerably higher than in the other two years (Fig 4). This increment was due to the presence of a higher number of Anthoathecata and Leptothecata hydromedusae species that are characterized by having a benthic stage (polyp) in their life cycle. It is known that higher water temperatures promote faster life cycles and higher reproductive rates [2,24]. The sea warming trend recorded during the studied period (Fig 2) may help to explain this result; higher temperatures would trigger the earlier release of some species of medusae from their polyp, thus favoring a higher number of species in the plankton realm [58]. However, because the increase in the number of species in 2011 was mostly caused by species with only one individual presence, and most of them were not collected in 2004 when the temperature was higher (Table 2), we cannot conclude that the temperature increase was the only factor influencing the species richness increment observed in 2011.

Changes in the spatial distribution pattern of the community observed among the surveys were based on the influence of environmental factors (Table 5). In the coldest survey, 1983, the community showed a clear coast-offshore ordination pattern, while in the warmer 2004 and 2011 surveys, a north-south pattern was noticeable (Fig 10). This north-south pattern has also been observed in the area during exceptionally warm summer conditions [53], which could indicate that a latitudinal ordination pattern of the planktonic cnidarian community will become more evident under increasing temperature scenarios.

The changes in the abundance of the cnidarian community observed in the present study could also be influenced by a decrease in the abundance of predators and competitors, such as small pelagic, planktivorous fish. In the Mediterranean, fish catches are dominated by the small pelagic sardine and anchovy, and since the mid-1990s, their landings have shown progressive decreasing trends in the northwestern sector [59,60]. It is well known that many species of fish consume gelatinous zooplankton [61,62], and in the NW Mediterranean, large deep-sea fish have been shown to positively select siphonophores as prey [63]. Therefore, any reduction in these vertebrate competitors, whether due to climatic change or overfishing, may lead to a lower predation pressure on gelatinous zooplankton and a decrease in competition for food resources [64,65], which would favor an increase in the abundance of gelatinous zooplankton. Additionally, studies related to the long-term variations of zooplankton in the western Mediterranean [66,67] suggest that the total zooplankton biomass has not decreased in the last few decades, which would also support the increase in carnivorous gelatinous zooplankton.

The observed increase in planktonic cnidarians might be a response to both the climate and the anthropogenic changes that have occurred during the last few decades in the NW Mediterranean. This may imply difficulties for the recovery of certain pelagic fish stocks competing for the same food [62,68], but also an increased availability of gelatinous prey for other fish and vertebrates [63,69].

## Conclusion

Under the current climate change scenario, planktonic cnidarian communities in temperate regions will increase their total stock and will suffer changes in their species composition, latitudinal distribution and phenology. The abundances of warm-water species, like *M*. *kochii*, will be particularly favored and their spatial distributions widen. We suggest to use the abundance ratio *M*. *atlantica*:*M*. *kochii* as a proxy for measuring gelatinous zooplankton responses to ocean warming in temperate areas. Based on these conclusions, we strongly recommend the study and monitoring of mesoscale spatial areas in order to understand not only the long-term changes in gelatinous zooplankton abundances but also how these changes affect their spatial distributions.
